# A model of non-pharmacological intervention (Agorà model) on behavioural disorders in patients with Alzheimer’s disease

**DOI:** 10.1192/j.eurpsy.2021.1975

**Published:** 2021-08-13

**Authors:** G. Conte, I. Sinisi, F. Franza

**Affiliations:** 1 Il Filo Di Arianna, Social Cooperative, Venosa, Italy; 2 Sir, Psychiatric Rehabilitation Centre Villa dei Pini, Avellino, Italy

**Keywords:** Behavioural disorders, old age psychiatry, Burnout caregivers

## Abstract

**Introduction:**

Cognitive deficits, behavioral disorders, neuropsychiatric symptoms (BNS) are characteristics in Alzheimer’s disease (AD). Morover, elderly patients often take multiple medications for their several chronic health conditions. Shared decision making is essential to deprescribing unnecessary or harmful medications in older adults. For these reasons, it may be useful to develop multiple strategies intervention not pharmacologically based and to raise the living standards of the patients, the healthcare professionals and the relatives directly or indirectly involved.

**Objectives:**

To show application of the Agorà model in AD to improve the performance levels, to decrease the aggressive behaviours and wandering episodes.

**Methods:**

Twelve inpatients (79-95 ys) affected by AD, were included in our observational study, recruited in Social Cooperative “Il filo di Arianna”, We have applied in our patients the Agorà model (from the Gentlecare model).Were administered following scales: in inpatients: NPI; CDR, MMSE; in caregovers: CBI; at baseline (T0), after three (T1), six (T2) twelve months (T3). For statistical evaluation we used the EZAnalyze Version 3.0 software, on Excel.

**Results:**

At T0 all patients showed high levels of behavioral and aggression disorders. After T3 with Agorà Model, there has been a significant reduction of previous levels. In addition, an improvement in CBI data was observed in caregivers.

**Conclusions:**

The application of the Agorà model has triggered better performance levels in AD. Moreover, it determined a decrease of behavioural disorders, promoted higher levels of participation in the everyday care activities, improved family wellbeing and participation to the assistance activities, reduced health care professionals turnover and burnout levels.

**Disclosure:**

No significant relationships.

